# Differentiation of High-Fructose Corn Syrup Adulterated Kelulut Honey Using Physicochemical, Rheological, and Antibacterial Parameters

**DOI:** 10.3390/foods12081670

**Published:** 2023-04-17

**Authors:** Rinee Najwa Mohamat, Nur Rabiatul Adawiah Mohammad Noor, Yus Aniza Yusof, Suriana Sabri, Norhasnida Zawawi

**Affiliations:** 1 Department of Food Science, Faculty of Food Science and Technology, Universiti Putra Malaysia, Serdang 43400, Selangor, Malaysia; rineenajwamohamat@gmail.com; 2 Halal Products Research Institute, Universiti Putra Malaysia, Serdang 43400, Selangor, Malaysia; rabiatuladawiah@uitm.edu.my; 3 Faculty of Applied Sciences, Universiti Teknologi MARA, Cawangan Negeri Sembilan, Kampus Kuala Pilah, Kuala Pilah 72000, Negeri Sembilan, Malaysia; 4 Department of Process and Food Engineering, Faculty of Engineering, Universiti Putra Malaysia, Serdang 43400, Selangor, Malaysia; yus.aniza@upm.edu.my; 5 The Enzyme and Microbial Technology Research Centre, Faculty of Biotechnology and Biomolecular Science, Universiti Putra Malaysia, Serdang 43400, Selangor, Malaysia; suriana@upm.edu.my

**Keywords:** sugar adulteration, Kelulut stingless bee honey, high fructose corn syrup, physicochemical properties, antimicrobial properties, *Heterotrigona itama*

## Abstract

Kelulut (stingless bee) honey (KH) possesses a wide range of benefits for human consumption and can exhibit medical effects. Due to its high value, this premium honey is often adulterated with different types of cheaper sugars, causing low nutrients and potential food safety threats in the final product. This study aims to determine the physicochemical, rheological, and antibacterial properties of sugar-based adulterated KH from the stingless bee species *Heterotrigona itama.* Adulterated samples were prepared using pure honey mixed with different concentrations of high fructose corn syrup (HFCS), i.e., 10%, 20%, 30%, 40%, and 50%. Water activity, colour, total soluble solids, pH, turbidity, viscosity, and antimicrobial activity of KH were determined. In addition, the primary sugar composition (fructose, glucose, and trehalulose) was determined by high-performance liquid chromatography with evaporative light-scattering detection (HPLC-ELSD). This study shows that the increasing percentage of HFCS addition in the KH samples significantly increases (*p* < 0.05) the total soluble solids, colour, pH, turbidity, viscosity, glucose, and fructose content; meanwhile, the water activity and trehalulose were reduced significantly (*p* < 0.05). Antimicrobial activity against *S. aureus* was reduced significantly (*p* = 0.006) by an increased percentage of HFCS compared to Control. Antimicrobial activity against *P. aeruginosa* was also found to be reduced significantly but showed non-significant effect from an increased percentage of HFCS in honey (*p* = 0.413). The bacterium *S. aureus* was more vulnerable to treatment with honey from both Control and adulterated groups compared to *P. aeruginosa*. In conclusion, HFCS-adulterated KH and authentic KH can be differentiated using all the parameters investigated. These data are vital for the governing bodies to ensure that KH sold in the markets is free from HFCS adulteration.

## 1. Introduction

In Malaysia, stingless bees of *Trigona* spp. produce multiflora honey known as Kelulut honey (KH), named after the bees producing it, commonly called Kelulut [1]. KH is more diluted than other honey and is famous for its unique flavour with sour-like taste and smell [2]. Research showed that honey from *Heterotrigona itama* has shown excellent antioxidant properties and possesses high mineral content [3]. Traditional medicine practitioners have used KH due to the beliefs on its medicinal usefulness and value [4]. According to current regulations, no other compounds or additives can be added to honey sold as authentic honey [5]. However, honey with inexpensive sweeteners such as maltose syrup and fructose syrup added are the most typical forms of honey adulteration practiced by irresponsible sellers [6]. These adulteration processes lower its nutritional value and consequently adversely affect human health [7].

Adulteration of authentic honey with sugar may also change the physicochemical and biological properties of KH. The recent discovery of trehalulose as a distinctive biologically active disaccharide in KH, specifically from species *Geniotrigona thoracica* and *H. itama* is considered a potential marker to determine its authenticity [8]. Similarly, authentication of Manuka honey also uses specific marker compounds derived from the nectar and DNA derived from the pollen to determine its authenticity [9]. Adulteration of honey with products containing disaccharides can mimic the primary carbohydrate profile of honey [10].

In recent years, there has been an increasing interest in using honey as a medicinal agent due to its great effectiveness against certain antibiotic-resistant microorganisms. Customers who purchase adulterated items in the market as antibacterial agents for wounds and infections may place their trust in the pure honey, which is recognised to have antimicrobial characteristics since adulterated honey may have significantly lower antibacterial and other healing capabilities than pure honey. This research was conducted to differentiate the physicochemical and antimicrobial properties of authentic KH with HFCS adulterated KH stingless bee species, *H. itama.*

## 2. Materials and Method

### 2.1. Procurement and Preparation of Sample

Raw Kelulut honey (KH) from the species of *H. itama* was harvested and purchased from a beekeeper in Bahoney Farm, Kampung Rinching Hilir, Kajang, Selangor, Malaysia. The sample was collected using sterilised, dried, and sealed Schott glass bottles and stored in a refrigerator with a temperature range between 0 °C to 5 °C. High fructose corn syrup was purchased from a local supermarket in Kuala Lumpur and stored at room temperature. To prepare adulterated samples, authentic KH was mixed with different concentrations of high fructose corn syrups, i.e., 0%, 10%, 20%, 30%, 40%, and 50% (*w*/*w*).

### 2.2. Physicochemical Analysis

All physicochemical properties were analysed according to CODEX Official Methods of Analysis [11], Malaysia Standard, MS 2683: 2017: Kelulut (Stingless bee) honey—Specification [12] and harmonised methods of the International Honey Commission [13]. There were two samples of KH that went through all of the analysis. Pure KH was assigned in a honey with 0% of HFCS, while adulterated KH was assigned in a honey with a different concentration percentage of HFCS. All measurements were carried out in triplicate (*n* = 2 × 3).

#### 2.2.1. HPLC-ELSD Analysis

High-performance liquid chromatography (HPLC-Waters Alliance 2695 HPLC system, Waters™, Millford, MA, USA) with evaporative light-scattering detector (ELSD) was used to determine the composition of fructose, glucose, and trehalulose in the samples according to Malaysian Standard, MS 2683: 2017 [12] with modification. The column used was Waters Spherisorb Amino NH2 (4.0 × 250 mm, 5 μm). A KH sample of 2 g was mixed with 19 mL deionized water, and the mixture was transferred into 100 mL volumetric flask [13]. 25 mL of methanol was added, and deionized water was added up to 100 mL. The stock solution was homogenized by shaking the flask before being filtered into the vials through a syringe filter 0.22 μm. The isocratic method was used with the mobile phase of 85% acetonitrile and 15% deionized water (*v*/*v*). The solution was filtered through a 0.45μm membrane filter and sonicated for 10 min before being used. The column temperature was set at 27 °C with a flow rate of 0.8 mL/min, and the ELSD detector was operated at 65 °C with pressure at 2.2 bar. The sugars were identified based on their retention times by comparing with sugar standards. The sugar concentration was calculated by using the calibration curve of each sugar.

#### 2.2.2. Water Activity (a_w_), Total Soluble Solids, pH, Colour and Turbidity

A small aliquot of honey (1 g) sample was used to determine the water activity by using an Aqualab water activity meter (Decagon Devices, Inc., Pullman, WA, USA) at 20 °C.

The total soluble solid of the sample was analysed using a handheld manual refractometer (Master-3M, ATAGO, Minato-ku, Tokyo, Japan) at 25 °C. 

Each sample with a concentration of 10% (*w*/*v*) in distilled water was analysed with a pH meter (Desktop pH meter Seven Compact S220, Mettler Toledo, Chiyoda, Japan) at a temperature of 25 °C [1].

The colour of the honey was analysed using a chromameter (Konica Minolta Chroma Meter, model CR-400, Osaka, Japan) by measuring the values of L*, a*, and b*. The colour of the adulterated Kelulut honey was determined by measuring the values of values of L*, a*, and b*. L* values indicate the brightness level, a* indicates red to green, meanwhile, b* indicates yellow to blue.

The turbidity of the samples was measured with a portable turbidimeter (Orbeco-Hellige Model 966, Sarasota, FL, USA), and the reading was stated in Nephelometric Turbidity Unit (NTU).

#### 2.2.3. Viscosity

The viscosity of the samples was determined by using a rheometer (AR G2 rheometer, Yumpu, Diepoldsau, Switzerland) using a parallel plate with a diameter of 40 mm for every sample. The gap between the plates was set to 0.5 mm. All analyses were conducted at 25 °C with the range of shear rate set from 0.1 to 100 s^−1^. The apparent viscosity was determined as a function of shear rate. A graph of viscosity (Pa.s) versus shear rate (1/s) and graph of shear stress (Pa) versus shear rate (1/s) was plotted.

### 2.3. Minimum Inhibitory Concentration (MIC)

Antimicrobial activity of adulterated honey samples was determined against *Pseudomonas aeruginosa* and *Staphylococcus aureus* isolates taken from The Enzyme and Microbial Technology Research Centre (EmTECH) Faculty of Biotechnology, Universiti Putra Malaysia, Serdang Selangor.

Bacteria were subcultured on tryptic soy agar (TSA) for 18 to 24 h at 37 °C. Three colonies were collected from the overnight plates using a sterile inoculating wire loop, and the bacteria were transferred to a sterile tube containing 10 mL of tryptic soy broth (TSB). The bacteria were then cultured overnight at 37 °C. The MIC of honey was determined using a modified version of the method used by Tuksitha, L. (2018) [14]. The inoculum was then diluted by diluting 0.05 mL of the modified inocula with 9.95 mL of TSB to a cell density of 5 × 10^5^ cfu/mL [15]. Each honey was converted into a stock solution (50% *v*/*v*) by weighing 5 mL of each honey and adding it to 10 mL of TSB. Using sterile TSB, the optical density (OD) of the bacteria suspension was adjusted to 0.5 McFarland (1–2 × 10^8^ cfu/mL) at 600 nm (Clinical and Laboratory Standards Institute/NCCLS 2012).

For each sample, serial dilutions were then produced to yield honey concentrations of 5%, 10%, 15%, 20%, 25%, 30%, and 50%. Each test well had a final volume of 200 μL, with three replicates per dilution, each consisting of 190 μL of each honey dilution and 10 μL bacterium inoculum. Several control wells were included in each assay: (1) wells containing 200 μL TSB only (without honey and inoculum), which served as the assay broth sterility control; (2) wells containing 190 μL TS broth and 10 μL inoculum (without honey), which served as the viability control; and (3) wells containing 200 μL honey dilution with TS broth (without inoculum), which served as dilution sterility controls. Overnight incubation was performed on 96 well flat-bottom microtitre plates in a shaker incubator at 120 rpm at 37 °C, and the absorbance of the wells was measured at 600 nm the following day. The following formula was used to compute the percentage suppression of bacteria growth for each honey dilution:

$$\frac{1-\left(\mathrm{Absorbance}\;\mathrm{of}\;\mathrm{test}\;\mathrm{well}–\mathrm{Absorbance}\;\mathrm{of}\;\mathrm{dilution}\;\mathrm{sterility}\;\mathrm{control}\;\mathrm{well}\right)}{\left(\mathrm{Absorbance}\;\mathrm{of}\;\mathrm{assay}\;\mathrm{viability}\;\mathrm{control}–\mathrm{Absorbance}\;\mathrm{of}\;\mathrm{broth}\;\mathrm{sterility}\;\mathrm{control}\right)}\times 100$$

The percent inhibition has a minimum value of 0% and a maximum value of 100%.

### 2.4. Statistical Analysis

All the experiments were performed in triplicates except for sugar analysis in duplicates. The data were analysed using the computational software of Minitab 19 (Minitab Ltd., Coventry, UK) and Microsoft Excel 2016 (Microsoft, Redmond, Washington USA). The data tested were normally distributed and one-way analysis of variance (ANOVA) and Tukey Pairwise Comparison were performed to determine the mean difference and any significant differences between the sample mean at (*p* < 0.05). The results of the analysis were presented as mean ± SD (standard deviation) of triplicates.

## 3. Results and Discussion

All physicochemical properties of adulterated KH with HFCS at different percentages were determined. Sugar analysis was carried out in duplicate (*n* = 2 × 2), and the rest of the analyses were carried out in triplicate (*n* = 2 × 3). The results of the analysis are presented in Table 1. 

### 3.1. Physicochemical Properties

Main sugar compositions in adulterated KH obtained from HPLC-ELSD analysis is provided in Table 1. For each sugar, linear regression was conducted producing linear equations as follows; y=499301x−532357 (R^2^ = 0.9977), y=518226x−815764 (R^2^ = 0.9995) and y=288093x−145393 (R^2^ = 0.9918) for fructose, glucose, and trehalulose, respectively. The R^2^ values obtained for the standard curve of each sugar are greater than 0.99 (R^2^ ˃ 0.99), which indicates a linear relationship between chromatographic linear response areas with the concentrations of all compounds (Yilmaz et al., 2014). The fructose and glucose content increased, while the trehalulose content reduced significantly (*p* ≤ 0.05) as the level of HFCS added increased. The fructose, glucose, and trehalulose content ranged between 14.15 to 31.97 g 100 g^−1^, 14.01 to 25.11 g 100g^−1^, and 12.41 to 24.11 g 100g^−1^, respectively. Recently, trehalulose was discovered as the main sugar composition in stingless bee honey for the first time through research conducted on honey from different species of stingless bees from Malaysia, Australia, and Brazil [8]. Trehalulose is a natural structural sucrose isomer that releases monosaccharides into the bloodstream slowly and gradually compared to sucrose [20]. The addition of HFCS increases the amount of fructose and the glucose content, which can be explained by the composition of HFCS, which primarily contains the monosaccharides glucose and fructose [21].

The range of water activity in the samples was between 0.686 to 0.761 as presented in Table 1. The water activity of KH decreased as the percentage of HFCS added increased (*p* ≤ 0.05). Water activity refers to the “ratio of the vapour pressure of water in a system to the vapour pressure of pure water at the same temperature” [22]. Generally, KH has higher water activity compared to *Apis* spp. Honey [23]. Previous research has observed changes in water activity associated with the adulteration of HFCS at different levels [24]. Another research study conducted to evaluate adulteration of fructose and hydrolysed inulin syrup in honey samples showed that as the level of the adulteration agent increased, the water activity increased proportionally [25]. 

Table 1 also shows that the total soluble solids in the samples increased significantly with the increasing amount of HFCS adulterated in the samples (*p* ≤ 0.05). Total soluble solid is one of the ways to estimate sugar content and increasing the value of total soluble solid can be associated with increasing sweetness level [26]. Organic compounds such as acids and minerals are components that can also influence the total soluble solid in honey [27].

The pH value of the samples increased significantly with increasing HFCS (Table 1). The pH level is one of the significant parameters in honey for extraction method and the condition of product storage which influence the texture, stability, and shelf life [28]. A study on the impact of blossom honey adulteration with HFCS at different concentrations from 0% to 100% showed a significant increase in the pH values of the honey [29]. The same trend was observed in research on Pakistani honey, in which the pH values of adulterated honey with sugar syrup were greater than pure honey [30].

The colour of the adulterated KH was determined by measuring L*, a*, and b* values. The increasing amount of HFCS added into the KH significantly increased the L*, a*, and b* values (*p* < 0.05) as presented in Table 1. The highest L* value was obtained from 50% HFCS, whereas the lowest was 0% HFCS with L* values of 40.123 and 32.127, respectively. As for a* values, 50% HFCS showed the highest value of 16.920 and the lowest value of 12.410 of 0% HFCS added into KH. The same trend was observed on b* values, ranging between 7.590 (0% HFCS) and 19.407 (50% HFCS). L*, a*, and b* values for KH from *H. itama* were 24.90, 1.90, and 2.52, respectively [23]. Colour is one of the indicators for the quality deterioration indicator of honey during storage and is affected by moisture content and storage temperature [31]. Previous research reported that colour analysis shows that authentic honey contains more red components (more reddish), whereas adulterated honey is usually luminous [32].

Turbidity (presented in NTU: Nephelometric Turbidity Units) for each sample with different amounts of HFCS adulteration ranged between 11.533 to 92.770. Table 1 shows that the increasing value of HFCS added in the samples significantly increased the value of NTU as indicated by *p*-value (*p* ≤ 0.05). A total of 50% HFCS adulteration had the highest turbidity (92.77) compared to other levels of adulteration. Turbidity is a parameter that can indicate the crystallisation of honey [33]. The crystallisation process in honey can influence customers’ preferences by lowering their acceptability [34]. The increasing percentage of glucose can explain the increasing turbidity values due to the strong effect of glucose on the crystallisation of honey [35].

**Figure 1:**
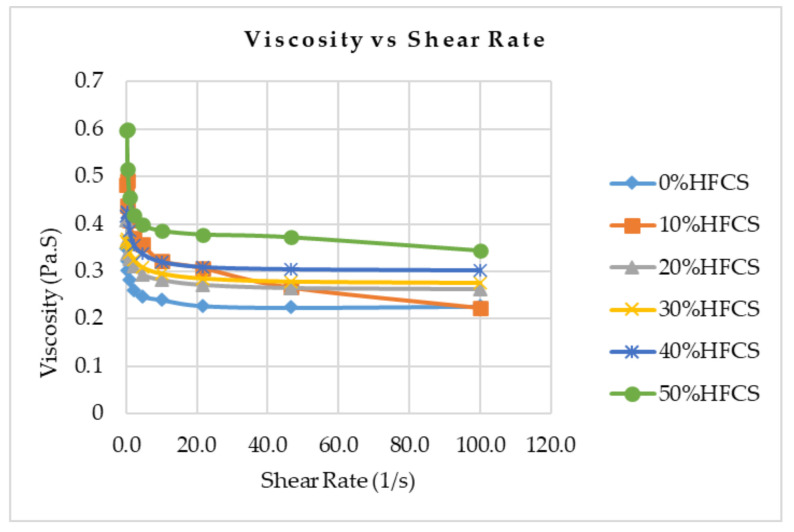
Effect of HFCS adulteration on the viscosity of adulterated KH at a different shear rate.

Figure 1 shows the impact of high fructose corn syrup adulteration on KH at a different percentage at a constant temperature of 25 °C. For each sample, the viscosity increased with increasing shear rate. This result can be associated with decreasing water content contributed by the adulterants added as viscosity is influenced by the flow behaviour [36]. The viscosity of HFCS (55% fructose) at 25 °C was between 0.212 Pa.s to 0.700 Pa.s [17,37]. The viscosity of the syrup was varied and affected by the composition of fructose and glucose and comparable at the same solid content. A high concentration of fructose results in lower viscosity, whereas a high glucose concentration, will increase the viscosity. Different shear rates significantly affected the viscosity of the sample with the *p*-value of 0.00 (*p* ≤ 0.05).

**Figure 2:**
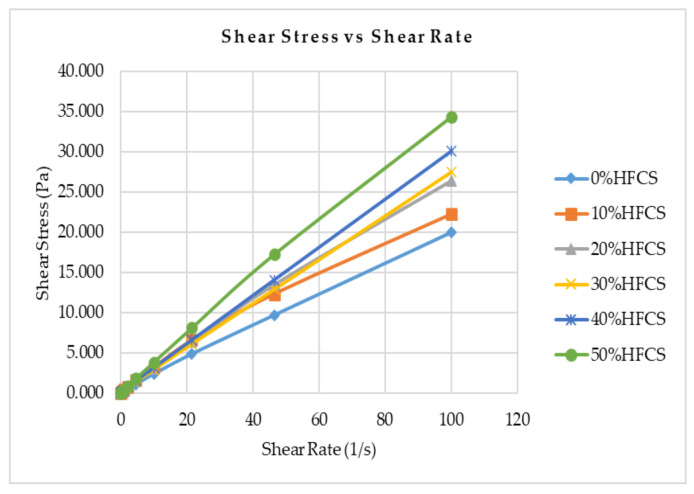
Correlation between shear stress with a shear rate on adulterated KH.

This result supports the correlation between shear stress and shear rate data, as shown in Figure 2. The graph indicates that the shear stress increased linearly with the shear rate, which indicates that the honey samples were having Newtonian flow behaviour [38,39,40]. The result from two-way ANOVA showed a significant interaction between the effects of shear rate and sample (formulation) on shear stress of the samples (*p* ≤ 0.05). The *p*-value of shear rate was 0.00 showing that different shear rate has a significant effect on the shear stress of the samples. The shear stress of the samples was also associated with different levels of HFCS added with *p*-value = 0.00. Another study suggested that the dynamic viscosity of adulterated honeydew honey is affected by the type of adulterating agent used. The results from the study proved that the dynamic viscosity declined with the addition of fructose but increased with glucose and hydrolysed inulin syrup [38].

### 3.2. Minimum Inhibitory Concentration (MIC)

**Figure 3:**
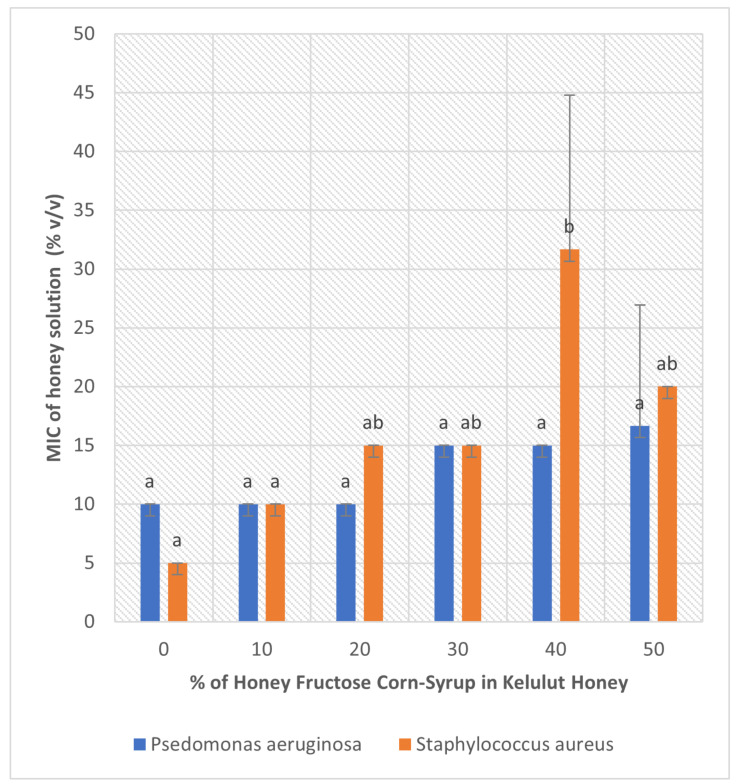
Antibacterial activity of adulterated Kelulut Honey samples of *H. itama* species with difference concentration HFCS against *P. aeruginosa* and *S. aureus*. Note: Means that do not share a letter are significantly different.

The antibacterial activity of the tested honey samples was expressed as MIC of honey samples produced by *H. itama* adulterated with difference concentration of honey fructose corn-syrup (HFCS) against two distinct bacteria: *P. aeruginosa* and *S. aureus* (Figure 3). The minimum inhibitory concentration (MIC) is the lowest quantity of substances required to inhibit bacterial growth [14]. As a result, a lower MIC value indicates greater antimicrobial activity.

There was no significant difference in effect of different HFCS-KH concentrations against *P. aeruginosa* in this investigation (*p* = 0.413). The antibacterial action of honey towards this bacterium species was unaffected by increasing HFCS concentration in KH. The lowest MIC value found in honey samples against *P. aeruginosa* was 10%, while the highest MIC value found was 25%. This finding is in line with Tukshita et al., 2018 [14] and Mat Ramlan, N.A.F., 2021 [15], who found that honey from the *H. itama* species had the lowest MIC against *P. aeruginosa*, at 10% (*w*/*w*).

Pure KH had the lowest MIC value (5%) when tested against *S. aureus*. Artificial KH at 40% HFCS concentration was the least effective against the organisms that were examined with MIC value 31% (*v*/*v*). *S. aureus* was more sensitive in pure KH compared to adulterated KH in this study. The MIC values of varied concentrations of HFCS-KH treated *S. aureus* showed significant variances (*p* = 0.006). The MIC values of varied concentrations of HFCS-KH treated *S. aureus* showed significant variances (*p* = 0.006). Increased MIC values were associated with increased concentration of HFCS, indicating increased loss of antibacterial activity. The MIC value of KH generated by *H. itama* against *S. aureus* was found to be lower than results reported in the study by Tukshita et al. (2018) [15], where the MIC was 10% *v*/*v*. A previous study by Mat Ramlan et al. (2021) [15] also reported reduced MIC values (6% *v*/*v*–8% *v*/*v*) against *S. aureus* in KH produced by *H. itama*.

In general, all the honeys tested had some activity against all the microorganisms tested, albeit to variable degrees of sensitivity. However, as the concentration of honey utilised was reduced, the antibacterial activity decreased [41]. The key reasons for the changes in antibacterial potential between pure and adulterated honey employed in this analysis could be the honey’s chemical profile due to HFCS concentration [42]. In this study, the different concentrations of HFCS were added to KH sources of honey yielded significant difference in the physicochemical properties of the honey. For example, this study explained that colour is one of the quality deterioration indicators of honey during storage and is affected by moisture content and storage temperature. Physical examination found that the colour of the honey samples and the adulterated honey used in the study differed significantly and that the colour of the honey samples degraded as the HFCS quantity in honey increased. As a result, the antibacterial action against the studied bacteria was lowered, as was the efficacy of the exhibition. A study by Bugarova et al. [43] showed that raw honey and adulterated honey give different amounts of sugar level thus reflecting the antibacterial activity of honey. Adulterated honey will give lower antimicrobial activity compared to raw honey. This sugar would be completely absent or present in minute amounts in honey that has been processed [43]. Observations made in accordance with nearly every study comparing the antibacterial capabilities of authentic and contaminated honey revealed that the efficacy of the prevention of bacterial species growth decreases as the amount of adulterated honey is increased.

## 4. Conclusions

In this study, physicochemical qualities were used as the criterion to investigate the effect of adulteration of KH with different amount of HFCS. Significant differences were observed after KH honey was adulterated. It is recommended to develop effective, inexpensive, and time-saving methods to detect adulterated KH honey using these parameters in the future. This study also reported for the first time that *S. aureus* was found to be more sensitive than *P. aeruginosa* in detecting adulteration of HFCS in Kelulut honey. Thus, an antimicrobial study of Kelulut honey against the microbe *S. aureus* can be used as an indicator of HFCS-adulterated honey by governing bodies to curb the marketing of these products in the local and international market.

This study focuses only on direct adulteration, so further investigation of indirect adulteration on KH should also be pursued to ensure honey quality in the market. 

## Figures and Tables

**Table 1:** Physicochemical characteristics of Kelulut honey adulterated with HFCS at different percentage.

Sample		HFCS	0%	10%	20%	30%	40%	50%	*p*-Value
**Fructose**		55.0 [16,17]	14.15 ± 1.23 ^d^	16.39 ± 0.58 ^cd^	20.44 ± 0.57 ^bc^	21.28 ± 0.69 ^b^	23.56 ± 1.42 ^b^	31.97 ± 1.98 ^a^	0.00
**Glucose**		45.0 [16,17]	14.01 ± 0.95 ^d^	15.21 ± 0.49^cd^	17.76 ± 0.38 ^bc^	18.36 ± 0.32 ^bc^	19.48 ± 1.09 ^b^	25.11 ± 1.41 ^a^	0.00
**Trehalulose**		NA	24.11 ± 3.39 ^a^	20.63 ± 1.02 ^ab^	19.36 ± 0.48 ^abc^	14.08 ± 1.28 ^bc^	13.25 ± 3.27 ^bc^	12.41 ± 1.25 ^c^	0.002
**Water activity**		0.738 [18]	0.761 ± 0.007 ᵅ	0.740 ± 0.004 ᵇ	0.730 ± 0.011 ᵇᶜ	0.723 ± 0.004 ᶜ	0.697 ± 0.003 ᵈ	0.686 ± 0.001 ᵈ	0.00
**Total soluble solid**		75.6–77.0 [17,19]	69.067 ± 0.115 ᵈ	69.667 ± 0.231 ᶜ	70.00 ± 0.00 ᶜ	70.867 ± 0.231 ᵇ	71.733 ± 0.115 ᵅ	72.133 ± 0.058 ᵅ	0.00
**pH**		4.65–4.80 [19]	3.190 ± 0.00 ᵈ	3.223 ± 0.006 ᶜᵈ	3.243 ± 0.006 ᵇᶜ	3.257 ± 0.006 ᵅᵇᶜ	3.290 ± 0.044 ᵅᵇ	3.297 ± 0.006 ᵅ	0.00
**Colour**	L*	63.46–65.26 [19]	31.513 ± 0.218 ᵈ	32.127 ± 0.263 ᵈ	33.70 ± 1.89 ᶜᵈ	35.717 ± 0.142 ᵇᶜ	37.827 ± 0.392 ᵇ	40.123 ± 0.040 ᵅ	0.00
	a*	6.20–7.42 [19]	12.410 ± 0.616 ᵈ	12.723 ± 0.320 ᵈ	14.053 ± 0.760 ᶜ	15.513 ± 0.130 ᵇ	16.520 ± 0.240 ᵅᵇ	16.920 ± 0.339 ᵅ	0.00
	b*	26.87–29.47 [19]	7.590 ± 0.370 ^e^	8.300 ± 0.208 ^e^	9.903 ± 0.620 ᵈ	14.280 ± 0.104 ᶜ	16.103 ± 0.323 ᵇ	19.407 ± 0.425 ᵅ	0.00
**Turbidity**		NA	15.967 ± 0.416 ᵈ	11.533 ± 0.306 ^f^	13.867 ± 0.379 ^e^	26.433 ± 1.305 ᶜ	38.533 ± 0.643 ᵇ	92.77 ± 0.208 ᵅ	0.00

Note: The data expressed as mean ± S.D. (*n* = 2); the different letters within the rows indicate statistically significant differences determined using ANOVA (*p* < 0.05).

## Data Availability

The data presented in this study are available.
